# Reply

**DOI:** 10.1016/j.jvscit.2021.04.007

**Published:** 2021-05-03

**Authors:** Shuhei Yoshida, Isao Koshima, Hirofumi Imai, Ayano Sasaki, Shogo Nagamatsu, Kazunori Yokota

**Affiliations:** International Center for Lymphedema, Hiroshima University Hospital, Hiroshima, Japan; Department of Plastic and Reconstructive Surgery, Hiroshima University, Hiroshima, Japan

We are grateful for the opportunity to respond to the letter to the editor by Norimatsu and Norimatsu regarding our article, “Lymphaticovenular anastomosis for recurrent cellulitis in a dementia patient with lymphedema,”^1^ which described a case of lymphedema in which compression therapy could not be used because of the patient's dementia. Referring to their study,^2^ Norimatsu and Norimatsu suggested the possibility that malnutrition, hypertension, and hyperlipidemia might have contributed to the recurrent cellulitis accompanying lymphedema in our patient.

During the treatment period, our patient had had a body mass index of 23 kg/m^2^. The low-density lipoprotein cholesterol level was 111 to 135 mg/dL, and the systolic/diastolic blood pressure was 90/50 to 130/70 mm Hg. A loss of appetite was not observed. However, the albumin level was only slightly low at 3.0 to 3.6 g/dL. We cannot deny that hypoalbuminemia might have contributed to the recurrence or worsening of cellulitis, and we must acknowledge that the presence of hypoalbuminemia is an unresolved factor. We planned to monitor the patient's albumin level carefully and to optimize her nutritional status to help prevent the future recurrence of cellulitis. However, the hypoalbuminemia had persisted after the recurrent cellulitis had resolved. Thus, we believe that the hypoalbuminemia was not the main cause of cellulitis but one of the causes. Recurrent cellulitis can be attributable to many factors, including aging.

Recent studies have shown that in elderly people with recurrent cellulitis, aging lymphatic collecting ducts have decreased contraction frequency,^3^ decreased systolic lymph flow velocity and pumping activity,^4^ and decreased lymphatic vessel density and complexity.^5^ Clinical studies using lymphoscintigraphy^6^ and indocyanine green fluorescence lymphography^7^ have also demonstrated a reduction of lymph drainage with increasing age. In addition, our previous studies revealed that lymphatic vessel transport tended to be delayed in older patients^8^ and that lymphaticovenular anastomosis is effective even in older patients with lymphedema.^9^ We believe that, in addition to maintaining the albumin levels, maintaining lymphatic transport is important because it plays a key role in the immune function in the limbs.
